# Integrating Google Maps and Smooth Street View Videos for Route Planning

**DOI:** 10.3390/jimaging11080251

**Published:** 2025-07-25

**Authors:** Federica Massimi, Antonio Tedeschi, Kalapraveen Bagadi, Francesco Benedetto

**Affiliations:** 1 Signal Processing for Telecommunications and Economics Lab., University of Roma Tre, Via Silvio d’Amico 77, 00145 Rome, Italy; 2 School of Electronics Engineering (SENSE), VIT-AP University, Amaravati 522 241, Andhra Pradesh, India; kalapraveen.b@vitap.ac.in

**Keywords:** car detection, google street view, image recognition, lane detection, route planning, scenic video

## Abstract

This research addresses the long-standing dependence on printed maps for navigation and highlights the limitations of existing digital services like Google Street View and Google Street View Player in providing comprehensive solutions for route analysis and understanding. The absence of a systematic approach to route analysis, issues related to insufficient street view images, and the lack of proper image mapping for desired roads remain unaddressed by current applications, which are predominantly client-based. In response, we propose an innovative automatic system designed to generate videos depicting road routes between two geographic locations. The system calculates and presents the route conventionally, emphasizing the path on a two-dimensional representation, and in a multimedia format. A prototype is developed based on a cloud-based client–server architecture, featuring three core modules: frames acquisition, frames analysis and elaboration, and the persistence of metadata information and computed videos. The tests, encompassing both real-world and synthetic scenarios, have produced promising results, showcasing the efficiency of our system. By providing users with a real and immersive understanding of requested routes, our approach fills a crucial gap in existing navigation solutions. This research contributes to the advancement of route planning technologies, offering a comprehensive and user-friendly system that leverages cloud computing and multimedia visualization for an enhanced navigation experience.

## 1. Introduction

Recent technological innovations have enabled the translation of many aspects of our lives, originally thought for a paper world, into digital services migrated and extended in the cloud universe [1]. For centuries, the task of reaching an unknown location has been realized via the consultation of printed maps, showing the related road area’s representation. Maps can be precise and detailed, but, as schematic and two-dimensional projections, they do not facilitate a traveler who is unaware of the area. Today, the digital replica of this service has been enriched with a wealth of additive information, including satellite images and the automatic creation of routes. These two value-added services offer significant advantages in terms of awareness of the region and of its facilities. The possibility of examining route directions via satellite images offers the user a deep and comprehensive knowledge about the location, as well as the advantage of studying the path in advance.

In the last years, some interesting and innovative digital services have emerged, such as Google Street View [2], allowing seeing roads’ pictures simultaneously along with schematic route directions visualization. Through this service, a user can easily match the two pieces of information for a better understanding of the route and its surroundings (i.e., shops; road pavement defects). However, this kind of analysis requires manual viewing and considering a huge number of images, even for short-length paths, becoming a very time-consuming operation. If the user wants to see the full route, he/she must spend too much time on this service and might end up abandoning it for other online solutions (e.g., video made by travelers on YouTube or travel and adventure blogs for touristic destinations). Usually, and to save time, some users tend to select only the images of crucial points (e.g., road crossings, turns, etc.) in the itinerary. In addition, some images are not present in the image collection provided by the service, or they do not meet the user’s needs for some reason. For example, a route can be traversed in both driving directions but has been mapped only in one way by the Google Street View operator.

The use of images in road fields led to the increased use of computer vision (CV), i.e., a set of techniques that allows one to understand some or all of the information contained within a photographic or video resource. CV has risen in popularity in the technological sector over the past decade thanks to the self-driving car research field, which led to the definition of machine learning (ML) and deep learning (DL) models for object recognition tasks. The most employed techniques in this field are lane and car detection which aim to identify, respectively, the lines belonging to the road’s lanes and the vehicles currently driving on the roads. Nowadays several applications exploit these two CV techniques, for instance (i) the automatic traffic control consisting of a dynamic traffic estimation through the analysis of the cameras [3]; (ii) the vehicle detection, involving the automatic identification and monitoring of a vehicle in a road. In particular, the work in [4] describes how it is possible to track vehicles (with limited visibility) by merging vehicle parts from multiple cameras, while in [5] DL models and decision trees are exploited for monitoring traffic. Furthermore, video processing techniques are also known to be very effective in traffic speed control (TSC) applications [6].

In the last decade, some applications have been developed to help users explore and understand route directions by combining Google Maps services [7] with image processing and video creation techniques. For example, the Google Street View Player application (also named Hyperlapse) [8] allows users to create a video of a road trip in which the images change quickly, as taken from a camera placed in front of the car. This tool creates an extremely fast time-lapse through a simple JavaScript application running on our browser. Hyperlapse offers a highly dynamic video of the road oriented towards a single geographic point. The video is therefore characterized by an orientation guided by a focal point, fixed for the entire journey. Another web application is Gaiagi Driving Simulator [9] which provides driving experience by defining different road views, such as the route direction on a 2D map, 3D views, street view, and video sequence. These two applications are based on Google Maps services for getting route directions and street view images for creating a time lapse or video sequences of the route. However, none of them represents a proper solution for the needs of the user in the analysis and understanding of a route. In addition, Hyperlapse has the problem that the images are not put back in the direction of the user’s motion. Hence, the visualization of bridges or roads in the opposite direction of the travel can happen. Moreover, they do not address the issues related to the lack of images in the street view or the wrong image mapping of the desired road, and they are exclusively client-based applications.

Addressing some of these issues, we propose an automatic system that generates videos of road routes between two geographic locations. Given the endpoints of the path, respectively called the start and end, the system calculates the path, showing it both in the classical way (i.e., highlighting the track on a two-dimensional representation) and in a multimedia fashion. We develop a prototype on a cloud-based client–server architecture divided into three modules: (*i*) frames acquisition; (*ii*) frames analysis and elaboration; and (*iii*) persistence of the metadata information and computed videos. In particular, the frames acquisition module collaborates with two services offered by Google Maps [10] to calculate the route (Google Directions API [11]) and retrieve road panoramas (Google Street View Static API [12]). This module, like the persistence one, is written in C# by defining a typical client–server application based on the REST paradigm and common design patterns. The frames analysis and elaboration module, instead, has been written in C++ for high-performance computing requirements and exploits the OpenCV library [13], known for its potential in the field of CV and image processing. This module is invoked by the server once the first module has acquired the target route panoramas. Finally, we save the metadata of the images, the created paths, and the reference to the blobs of the processed videos to have them already available at any moment without creating them again.

The remainder of this work is organized as follows. Section 2 provides a deep review of the current state of the art for each of the features developed in our system and highlights the differences between our product and similar solutions. Section 3 details our system, analyzing the chosen workflow and architecture in terms of material and methods, discussing the two main modules of the system: frame acquisition and frame analysis and elaboration. Section 4 contains the results generated by our client–server system. Finally, conclusions are briefly outlined in Section 5, while existing limitations and future directions of our work are illustrated in Section 6

## 2. Related Works

Several solutions for creating a video of the route path on the road have emerged in the literature. As a common substrate, they are based on the following steps:

*(i)*  Compute the route by setting a start and an endpoint by using Google Direction API, HERE Routing API [14], or another third-party API;
*(ii)*  Download the panoramas or thumbnails provided by Google Street View Static API or similar tools;
*(iii)*  Process them on the client side (e.g., a web browser or mobile device) or server side to crop the panorama and perform minor adjustments;
*(iv)*  Create a video sequence by using the processed panorama frames.

More specifically, in [15], a system is proposed that takes start and end points as inputs and automatically connects to Google Maps with Street View to achieve the route planned result and scenery along the route. However, this system has some limitations: sometimes the near panorama is not recorded correctly in the XML file, since the relationship between panoramic images is wrong, and the system is not able to generate reasonable outputs. Another problem caused by the original data is that the next image may have a distorted view for some unknown reasons, and this problem is not logged in its XML file. In this case, the alignment will be completely wrong due to the different viewing angles. This is probably due to errors in the original data obtained from the Google Street View service. Hence, using real data to build a useful system requires a lot of effort to correctly handle the original data. Cube2Video is another valuable application presented in [16] to support navigation between cubic panoramas in video display mode. To reach this goal, it addresses the difficult problem of discontinuity in cubic panoramas in a simple and unified way. A new angular error is proposed to improve matching accuracy. The temporal fluidity of the video is improved by exploiting the cube map hardware support and the parallel computing power of the GPU, thus obtaining a video display in real time. However, this method globally deforms the input images, thus inducing rendering artifacts [17]. Hyperlapse is an automatic generator of extremely fast time-lapse prototypes. Through this web client application, the user establishes the start and the end point of the route together with a third option, named travel mode, that identifies the point of view for the video generation. The produced output consists of an extremely fast video showing the selected route taken with the camera oriented toward the indicated point of interest. For the application purposes, it is convenient to indicate purely straight roads. The frames turn and change perspective, framing the sky, the parapet of bridges, and even roads (as shown in the rest of this paper, we mathematically find a solution for these problems). Gaiagi Driver 3D is a more sophisticated software than Hyperlapse, and it provides different kinds of views that simulate the driving behavior of the user from different perspectives helping the users to explore the surroundings of the route. These two applications exploit the services provided by Google, and they do not manage any data persistence or more complex operational flow through a typical client–server architecture. Moreover, both applications do not address issues related to the wrong direction. As a matter of fact, sometimes the output video is composed of frames in the opposite traveling direction without informing the final user about the issue.

In this work, the aforementioned issues are considered and solved together: first, we extrapolate the frames through processing on the polyline used by Google to represent the path on the road map, carrying out a further phase of interpolation and filtering on the obtained data. This type of technique is necessary since the frame granularity (i.e., the number of frames) is too low in correspondence of straights and too high in the presence of roads’ curves. In addition, it uses an ad hoc cropping of the panorama according to the road through refined processing of the frames, exploiting the 360-degree images (i.e., photospheres), and processing them with the cosine theorem. Having as a goal the simulation of a road guide, one of the objectives of our project is to be able to identify and report frames taken in the wrong direction. The created methodology requires the use of two techniques related to CV: lane and car detection (these techniques will be detailed in the next sections) to put all the frames back in the correct running order. To the best of the authors’ knowledge, there are no papers focusing on methods for wrong-way frame detection. However, recently two interesting papers [18,19] were published. The first one depicts a system for road surveillance to detect and track moving vehicle’s direction on two-way traffic roads from a CCTV viewpoint using deep learning, where the roadside noise is filtered out. The second paper illustrates a real-time intelligent transportation system (ITS) to detect vehicles traveling in the wrong way on the road using a custom dataset for training to create a deep learning model. Another example of improvement provided by our work is the utilization of the techniques collected under the area known as inbetweening. This technique is mostly used in the field of animation and cartoons as in [20], where a new framework is proposed to produce cartoon videos by retrieving color information from two input key frames following the animated movement guided by a sketch of the user. Then, in [21] a biomechanically constrained generative contradictory network is introduced to perform long-term inbetweening human movements, conditioned by the constraints of the keyframe. This method consists of increasing the number of available frames, thanks to several interpolation techniques. Furthermore, the process used by the applications involves the use of a collection of frames relating to the road, for which it is possible to carry out numerous analyses using CV techniques. In fact, in road environments, multiple CV methods can be used, such as car and lane detection. The applications of these two techniques in the road sector are endless. Not only is it possible to carry out vehicle tracking or traffic control, as already discussed in the previous section, but also to create intelligent cars capable of autonomous driving.

### 2.1. Car Detection

Car detection is defined as an object detection task that consists of the recognition of quadricycles such as cars. Several works focused their attention on the definition of vehicle detection methods in a video sequence to solve different tasks like vehicle tracking, vehicle classification, and traffic analysis. Chetouane et al. provide in [22] a deep understanding of the main vehicle detection methods tested under different weather and traffic conditions. Most of these techniques aim at detecting vehicles contained within each frame to determine the position and area occupied by them and to identify the side shown by the car, establishing whether it is the rear or the front one.

The recognition of an object is an extremely complex goal, as recognition must be performed in several scenarios, often characterized by numerous elements (different from the object in question), and several variable factors must be considered, such as color, scale, and rotation. For this reason, ML models are used based on supervised classifiers. These kinds of models are trained or fine-tuned by using training and test sets that describe the location and the bounding box of each object we want to recognize in the image or in the video frames. In any ML training activity, the quality of the training and test sets greatly affect the effectiveness of the task. According to the chosen ML/DL model, the dataset to use for the training or fine-tuning operations can be built differently. For instance, in a modern DL model, the dataset is composed of a set of images representing objects to recognize, and it is formatted by using the COCO [23] or the Pascal VOC [24] dataset annotation formats. Other ML models, such as the cascade classifiers [25], require the definition of a pair of datasets, respectively, consisting of positive examples (i.e., what the system must recognize) and negative examples (i.e., what the system must discard). In detail, the positive examples enable the model to understand and recognize the main characteristics (i.e., features) that describe the objects, while the second set will instead be used to refine the model in the training process. Nowadays, DL models are defined for object detection tasks using modern frameworks like TensorFlow [26] or PyTorch [27]. In the field of vehicle detection, several works based on DL algorithms were presented. In [28], Rawat et al. proposed a novel architecture to detect and segregate different classes of cars using the You Only Look Once (YOLO) algorithms. The advantage of these methods is that they can detect objects based on predicted regions, and it is a network with fast training times. A real-time car detection and safety alarm system is described in [29]. The authors present an intelligent system for safety warning when driving the vehicle based on a real-time support vector machine. Two functions have been considered, lane departure warning and forward collision warning. A fuzzy early warning controller was used in the collision avoidance system. In [30], the authors define a classification process for video-based vehicle detection with convolutional neural network (CNN) architectures. However, this type of experimentation has some limitations: unfortunately, it is not always possible to access good hardware and software resources. Training on the CPU alone will take weeks to complete training for a single model. In fact, any deep learning research generally requires one or more powerful GPUs to complete the training process in less time than CPU training (hours). Finally, in [31] Aziz et al. focus their work on the results of the implementation of a vehicle detection algorithm for self-driving cars on the toll road. However, this algorithm is not very accurate in detecting and tracking the car when the image is too dark in night conditions. In fact, a method had to be added to modify the parameters during the day and night in an adaptive fashion.

As we have already said, DL-based solutions require more computational resources in both training and on-production environments to be efficient and have high real-time throughputs. A popular and very simple ML algorithm for performing object detection task is the Haar Feature-based Cascade [32] which is a very simple and fast detection algorithm that does not require high (and costly) computational resources like GPUs to perform its tasks. As a matter of fact, this algorithm stands out among others thanks to its greater effectiveness. It tends to produce more false positive (FP) detections compared to modern DL solutions. This algorithm is used for many detection tasks such as vehicle detections and facial recognition. In [33], the Haar Feature-based Cascade and classifier are used for face detection in the presence of various image distortions such as blur, salty and peppery noise, changes in contrast, and brightness. In [34] the Haar classifiers are also used in algorithms of object detection entirely incorporated into a quadcopter UAV. In [35], Neumann et al. introduce a vehicle detection system based on a stereo camera system located behind the car’s front window. Stereoscopic computer vision is applied to locate objects in the environment, and subsequently different algorithms are evaluated by image-based pattern recognition. The work [36] proposes an innovative and effective digital video stabilization technique, which uses the features present in videos that often contain many facial features in the foreground such as the eyes, nose, and mouth. Haar-based object tracking improves the accuracy of motion estimation between two frames resulting in a smoother sequence after motion compensation. In [37], the authors focus specifically on the automatic car detection process. In fact, vehicle detection and counting are keys to reducing traffic congestion and increasing road safety by avoiding collisions between cars. Finally, in [38], in addition to the Haar classifier, SVM was also used.

In our work, we decided to use the Haar Feature-based Cascade Classifier for the detection of the front and rear of the cars to detect any wrong-way frames provided by Google Street View API. This choice has been motivated to obtain the best trade-off between the quality of detection and the computational resources required for the inference. The effectiveness of the proposed system hinges on a careful balance between performance, accuracy, and computational feasibility. One of the key architectural decisions was the adoption of the Haar Feature-based Cascade Classifier for vehicle detection. This choice was driven by its low computational overhead, fast inference speed, and seamless integration with our C++/OpenCV-based pipeline, which aligns with the design philosophy of creating a lightweight and easily deployable system. While Haar Cascades are known for their simplicity and real-time capabilities on modest hardware, we fully acknowledge their limitations. Specifically, they tend to produce a higher false positive rate compared to deep-learning-based detectors, and they lack robustness in complex or low-contrast scenes. As such, this approach represents a deliberate trade-off, prioritizing portability and computational economy over detection precision. That said, we recognize the rapid advancements in object detection architectures, particularly the emergence of YOLO (You Only Look Once) models, which have evolved significantly in terms of speed, accuracy, and training flexibility. Recent versions such as YOLOv7 through YOLOv12 offer substantial improvements in detection precision while maintaining real-time performance, especially when executed on GPU-equipped platforms.

### 2.2. Lane Detection

The term lane detection refers to the task of detecting lanes within a carriageway [39]. Its use can be intended for different purposes, such as highlighting the edges of lanes in an augmented reality application [40] or the understanding of the geometric model that describes the road to improve the safety of a self-driving car [41]. In the literature, there are different methodologies for road line detection. In [42], a robust method is proposed to overcome the various difficulties that arise in rainy weather via an Automatic Adjustable Threshold (AAT) in the Canny edge detector which improves its performance in the case of colored, eroded, and smudged road markings. However, despite being innovative, this project presents the following problems: it does not work well on curved roads, and if the car’s position is not centered, the lane tracking system cannot track the lane accurately. This type of problem is solved in [43] where a lane line detection method is proposed for intelligent vehicles in multiple road conditions. Dong et al. in [44] present a new lane deviation algorithm to detect lane markers in images captured by a vehicle-mounted front camera. The algorithm always considers the two lanes closest to the car and can detect the left and right lanes separately. These systems share a similar image-processing pipeline which is composed of three main stages: (i) frame pre-processing; (ii) filtering operation; (iii) frame post-processing

The frame pre-processing consists of identifying the lines describing the road considering each time a single frame. Having to analyze the information content, it is convenient to carry out pre-processing work on the incoming resource, filtering all the information that can alter the search or increase its associated cost. The associated values are in fact characterized by noise, as they are extracted as raw resources from analog instruments. The filtering operation on the image, called blur or smoothing, is therefore necessary. For this operation, a Gaussian filter can be used in this stage. Finally, the frame post-processing is mandatory because the processed product is not noise-free. The straight lines obtained do not actually represent the lines roads but instead represent any straight line passing through a contour detected by the Canny’s algorithm. So, this stage deals with the noise reduction carried out through the filtering of the lines. According to this main processing pipeline, we define a simple but efficient lane detection method employed in the wrong-way detection algorithm defined in this work. While several tools have emerged for route visualization using Google Street View data, such as Hyperlapse, Cube2Video, and Gaiagi Driving Simulator, our proposed system introduces critical architectural and functional innovations that set it apart from these existing solutions. First, unlike Hyperlapse and Gaiagi, which are purely client-side applications, our system is built on a cloud-based, modular client–server architecture. This design enables better scalability, persistent storage of metadata and processed videos, and integration with other services via RESTful APIs. Additionally, none of the existing tools implement automated error handling for typical issues encountered in Street View imagery, particularly wrong-way frame detection. Our approach incorporates a hybrid method based on geometric orientation estimation and computer vision techniques (i.e., car and lane detection) to reliably identify and discard frames misaligned with the intended travel direction. This significantly improves the visual consistency and realism of the generated videos. Finally, while tools like Cube2Video focus on panoramic continuity and real-time rendering, they often suffer from visual artifacts due to abrupt perspective changes. In contrast, our system applies custom inbetweening techniques—specifically frame blending and Laplacian pyramid interpolation—to create smoother transitions between frames, enhancing perceptual quality without introducing significant distortion. These distinctions, particularly in architecture, error handling, and frame processing, underscore the novelty of our approach and its practical advantages for immersive and intelligent route planning.

## 3. Materials and Methods

### 3.1. System Model

**Figure 1:**
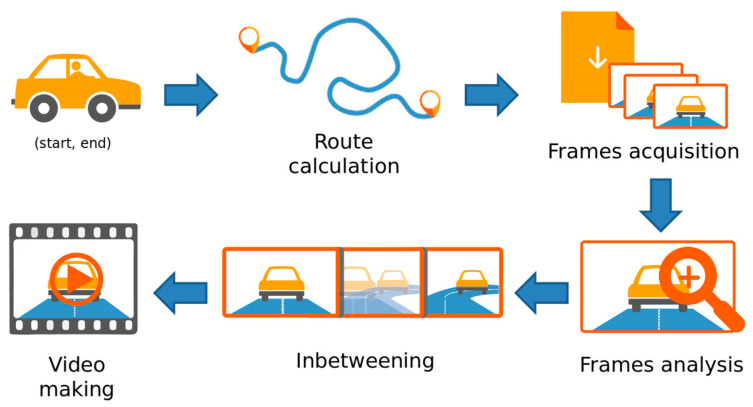
Workflow of the proposed system.

This section presents the pipeline and the main characteristics of our system. Given the complexity of the problem, we realize a prototype that includes the entire core of the application. We are interested in generating a video sequence that simulates the user driving his/her vehicle, therefore showing the route from his/her point of view. The user first uses a web UI to enter the start and end points of the route. Finally, the user receives the video in response through HTTP requests. From an initial analysis, it is essential to orient the camera so that it follows the movement of the virtual vehicle. In addition, the video frame rate must be reduced, making driving simulation less confusing and more natural for the end-user, who is then able to study the route. This operation requires the inclusion of smoothing flow techniques, collected in the area known as inbetweening, by creating intermediate frames between the main video keyframes. Therefore, the workflow of our system can be divided into two main blocks: one for frame acquisition and the other for frame processing and video making. The simplified workflow schema is shown in Figure 1.

The user enters the appropriate information regarding the path, which is interpreted by the system to generate the best route. Once calculated, the frames associated with the path are extrapolated, obtaining the entire needed set of images in a suitable orientation. Now, these frames must be processed to remove any artifact and/or error like missing and wrong-way frames. Finally, after the frames are interpolated with some inbetweening method, the video is created and ready to be analyzed by the user.

**Figure 2:**
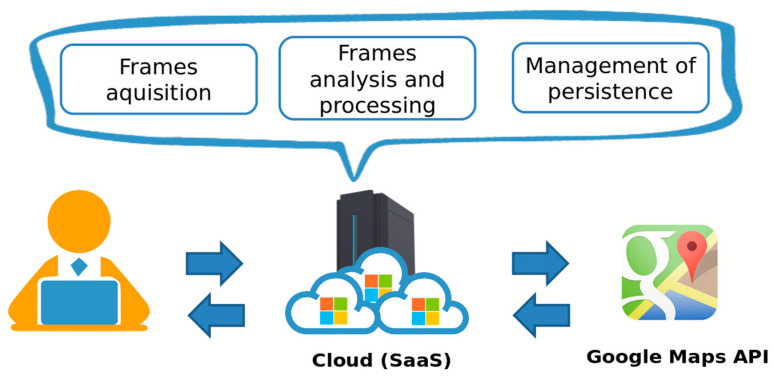
A simple representation of the cloud-based client–server prototype.

The choices made on the implementation of the system have guided us to the development of a client–server architecture based on the RESTful paradigm. It is destined for Cloud Computing services such as the Software as a Service (SaaS) type, as shown in Figure 2. This choice allows us to omit any aspect related to the selection and management of physical infrastructure, focusing our efforts exclusively on the creation of the software product. We decided to use the Microsoft Cloud Computing platform, known as Windows Azure, since it provides both Infrastructure as a Service (IaaS) and Platform as a Service (PaaS) service models. Moreover, it allows the creation of virtual machines (VMs) by choosing one of the available images and the VM computational resources (i.e., vCPU, memory, and storage). However, the solution can be easily adapted to any other public/private cloud provider thanks to its modularity. In fact, the architecture is realized through a modular approach, which has identified three main layers: frame acquisition, frame analysis, and processing and management of persistence.

The first module works closely with the Directions API and Static Street View API provided by Google Maps, retrieving information about the road and obtaining road 360-panoramas to process for creating the video. The second one deals with the analysis and processing of frames, exploiting image processing and CV techniques. We used a low-level language such as C++ to efficiently manipulate the images’ information using the OpenCV library. Finally, the metadata is saved. Given the large amount of possible information to be stored, MongoDB has been chosen as the database of our system. Through MongoDB’s flexible document schema, it is easy to evolve and store structured and unstructured data such as polyline and image metadata information. For our prototype, we created and configured on Microsoft Azure a MongoDB replica set system based on three Debian Linux hosts. Finally, to store the computed videos and the related processed frames, we use the object storage provided by Microsoft Azure, i.e., the Blob storage [45].

### 3.2. Route Definition and Frame Acquisition

Following the workflow presented in the previous section, here we present the frame acquisition module that deals with the first two tasks of our workflow: route definition and frame acquisition.

Given two coordinates, it is necessary to establish the itinerary that links each other. The proposed solution is to consider the polyline [46] reported by the Google Direction API for tracing the route on the map, extrapolating the points that constitute it. Due to the nature of the polyline, these points may turn out to be too dense for the cornering areas and too rarefied in the straight ones. Considering the purpose of the application, we are concerned with ensuring homogeneity in the granularity of information; therefore, the set of points is subjected to a suitable interpolation and filtering operation.

**Figure 3:**
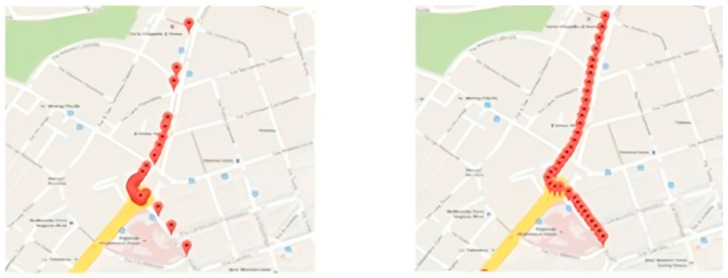
Coordinates points of (**left**) original Google Direction polyline and (**right**) post-processed Google Direction polyline.

First, the coordinates provided by the Direction API response, which describe the polyline, are extrapolated via a specific decoding function, and then the contents are interpolated with an interpolation function. This function has the task of bridging the distance between the pairs of coordinates, inserting one or more points, depending on the distance traveled between the two coordinates originally considered. The number of new elements depends not only on the initial gap between the pair but also on a threshold that guarantees a minimum distance, such that the request for the two associated images brings distinct results. Through interpolation and filtering on the various subsequences, a collection of coordinates at a homogeneous distance is obtained (see Figure 3).

**Figure 4:**
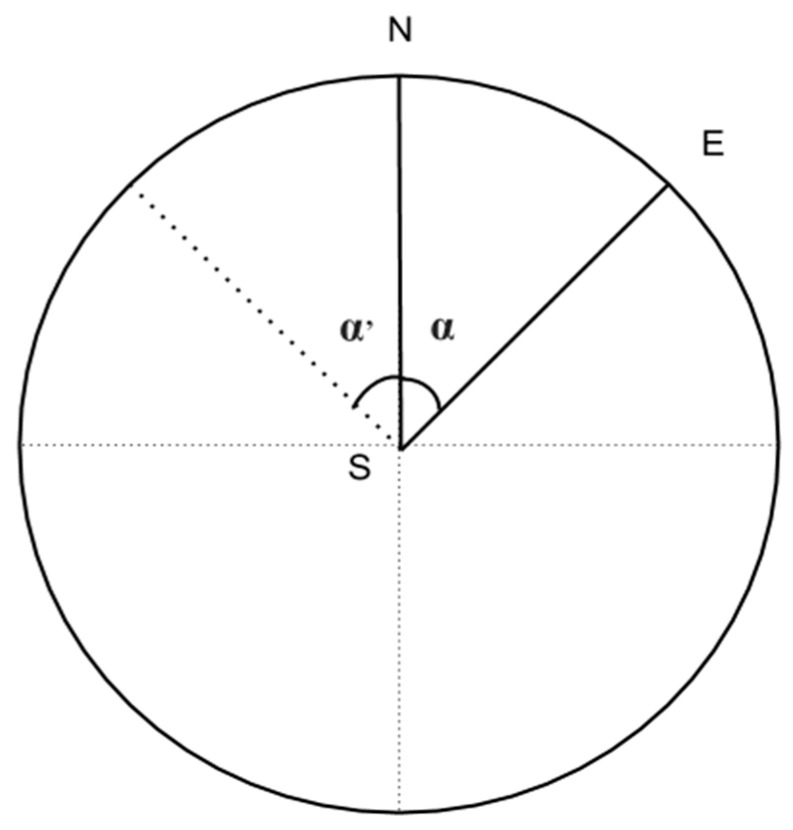
Circumference cosine theorem representation.

Once the software computes a homogeneous set of coordinates, it is now possible to extract a road picture for each of them. To create an immersive driving simulation experience for the user, the next frame must be oriented according to the previous coordinate’s frame. The use of Google Street View Static API [47] partially solves the problem related to the acquisition of the road picture, because it allows obtaining the 360-photosphere picture centered on given geographical coordinates. However, the photosphere pictures must now be processed before they can be used as frames in the video to avoid errors such as changes in visual perspectives, changes in altitude, etc. To process the 360-photosphere picture, it is possible to assume that each photosphere is composed of a set of frames projected onto a sphere. In particular, the provided algorithm selects the frame of the current photosphere which, seen from the center of the sphere, is oriented in the same direction as the next frame. This led our system to generate an automatic continuity effect in the final video. To perform this task, Google Static Street View gives the possibility to crop the area of a photosphere by using the angle with the Nord component. However, the angle calculation is our responsibility. Our solution is to address the issue mathematically, finding a formula that, given a pair of coordinates, determines the crop area through the angle with the Nord component. Our intuition is based on reducing the problem in a two-dimensional space as illustrated in Figure 4: it is possible to describe the set of coordinates, i.e., displacements, as a set of points on the plane, equidistant from each other, thanks to the homogeneity guaranteed by the previously described post-processing operations.

Considering the displacement characterized by the pair of coordinates S and E, shown in Figure 4, one may draw a circle of radius SE, which is the locus of points where S could be a result of the displacement performed. In accordance with the Google API, we simply find the angle α between SE and SN to know the exact location of the point E. The point N can be easily found by adding to the latitude S of the circle’s radius via the formula of the distance between two points. Once we have the description of the three points, we can apply the cosine theorem to find the angle:

(1) $$\underline{SE}=\sqrt{(E_{lat}-S_{lat}{)}^{2}+(E_{long}-S_{long}{)}^{2}}$$

(2) $$N=(S_{lat}+\underline{SE},S_{long})$$

(3) $$\underline{SN}=\underline{NE}$$

(4) $$\underline{NE}=\sqrt{(E_{lat}-N_{lat}{)}^{2}+(E_{long}-N_{long}{)}^{2}}$$

(5) $${\underline{NE}}^{2}={\underline{SN}}^{2}+{\underline{SE}}^{2}-2\underline{SN}*\underline{SE}\;cos(\alpha )$$

(6) $$\alpha =arccos(1-\frac{{\underline{NE}}^{2}}{2\underline{SE}})$$

Attention should be paid to (6), in that it determines the angle inside the triangle SNE, and therefore it is limited to 180°. To solve the problem, we simply check if the triangle affects α or α′ through the comparison between the coordinates (see Figure 4): it affects α′ if the longitude of E is smaller than the longitude of S. In this case, to evaluate the correct angle, it is sufficient to add 180° to the obtained value.

### 3.3. Frame Analysis and Processing

According to the system architecture described in the previous sections, it is now necessary to realize the second layer, i.e., the core of the system. It is characterized by three tasks: (i) inbetweening (frames interpolation); (ii) frames analysis; and (iii) frames processing via CV algorithms.

#### 3.3.1. Inbetweening (Frames Interpolation)

Despite the efforts described in the previous section for improving the frames’ granularity, the latter is not sufficient for the reproduction of the video to be perceived as smooth and acceptable by the user. In fact, the distance between two adjacent frames may be too high, and it is therefore advisable to interpolate the sequence for generating unrecovered frames or images that can simulate them. To solve this issue, we define the interpolation between two frames to generate a new one (NewFrame) which is a combination of these two frames and is placed between them. The available frames represent significantly changing landscapes, especially if they depict scenarios in highly congested urban environments. The product of the two generates an interpolated frame that is not clean and rich in elements and is not suitable for the generation of subsequent frames. In fact, given the amount of information contained, the new frames would add a perceptible noise, not respecting the chosen purposes. To solve such issues, our system exploits two popular inbetweening techniques, namely frame blending (see the pseudocode in Algorithm 1) and the Laplace pyramid (see the pseudocode in Algorithm 2) [48]. Through these two techniques, it was possible to generate a smoother and less fragmented animation composed of one or more intermediate frames starting from the key frames. Thanks to frame blending, we obtain a succession of images (i.e., intra-frames) always consisting of all the elements referring to the key frames. The frame blending technique consists of generating one or more frames by adding a crossfade between each real frame. Alpha blending is a computer graphics technique that simulates translucency: in addition to a red, green, and blue color, each pixel has a transparency component known as alpha. In this project the alpha percentage present in the frames is characterized by a factor of 50%. Through this assumption, the elements of a single frame are shown with less relevance and without understanding if they are present in both the original frames. Our simple implementation of the frame blender function is provided in the following pseudocode function.


**Algorithm 1.** Pseudocode of the frame blending technique employed in our work.frameBlender(prev, next) **for** (i in prev.rows) **do**   **for** (j in prev.cols) **do**    redPrev = prev.getRed(i, j)    greenPrev = prev.getGreen(i, j)    bluePrev = prev.getBlue(i, j)     redNext = next.getRed(i, j)    greenNext = next.getGreen(i, j)    blueNext = next.getBlue(i, j)     redNew = (redPrev + redNext)/2    greenNew = (greenPrev + greenNext)/2    blueNew = (bluePrev + blueNext)/2     newframe.setPixel(i, j, redNew, greenNew, BlueNew)   **end for** **end for** return newFrame


The function frameBlender receives inputs from two frames, i.e., prev and next, and it combines the RGB color matrices of the two frames to compute the new RGB matrices of the new frame which is returned by the function.

Then, the Laplace pyramid provides both noise cleaning and an increase in the resolution of the image but at the cost of a more complex algorithm. It is possible to identify a first phase where the creation of the two required data structures and their initialization takes place. This is realized by duplicating both frames in level 0 of the respective pyramid after having been suitably converted to grayscale. In fact, the information associated with the color is of no help during the analysis phase and can be re-entered at the end of the computation. Having set up level 0, it is possible to take care of subsequent levels, performing smoothing and subsampling operations on the base of the level previously worked, such that the i-th level has dimensions equal to half of the level (i − 1). We decided to use three levels for the pyramid. The pseudocode is shown in Algorithm 2.


**Algorithm 2.** Pseudocode of the 3-layer Laplace pyramid employed in our work.laplacePiramid(prev, next) pyramidLevel = 3 pyramidPrev[0] = rgb2grayScale(prev) pyramidNext[0] = rgb2grayScale(next) **for** (i = 1 in level) **do**    pyramidPrev[i] = downsample(pyramidPrev[i − 1])    smooth(pyramidNext[i])     pyramidPrev[i] = downsample(pyramidPrev[i − 1])    smooth(pyramidNext[i])
**end for** pyramidNew[lv]


After the first phase, the two structures are completed. It is therefore possible to proceed with the creation of a new pyramid, like the two previously defined, whose task is to keep the differences obtained by considering the images at each level. Then, the noise is reduced by applying smoothing operations. This operation allows for the creation of a blender for each pair of considered images and is like what was accomplished with the previously produced algorithm. Once the structure relating to the new frame has been obtained, it is necessary to reassemble the different available images within one, which exactly reflects the final product (see the pseudocode in Algorithm 3). The operations consist of carrying out the reverse procedure to what was implemented in the initialization phase. In fact, it is sufficient to iterate over the structure, adding the i-th level with the (i − 1)-th level, after having appropriately implemented upscaling operations on the resource with a smaller size (that is because the sum between images corresponds to a sum between matrices, which is possible only if they are the same size).


**Algorithm 3.** Pseudocode for reassembling the different available images within one.pyramidNew[lv] **for** (i = 0 in level) **do**    pyramidNew[i] = sub(pyramidPrev[i], pyramidNext[i])    smooth(pyramidNext[i])    **for** (i = level −1 to 1) **do**     upScaled = upscale(PyramidNew[i])     pyramidNew[i − 1] = pyramidNew[i − 1] + upScaled     **end for** **end for** gray2RGB(pyramidNew[0]) return pyramidNew[0]


**Figure 5:**
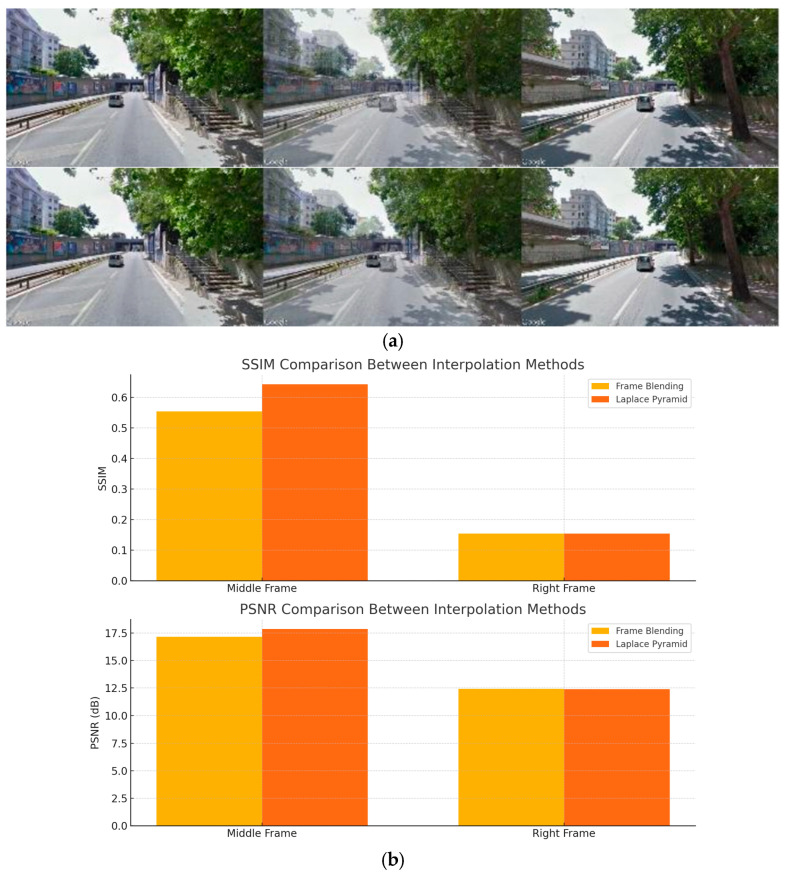
(**a**). Example of interpolation in an urban section, respectively, from left to right using (**upper**) the frame blending technique and (**lower**) the Laplace’s pyramid technique. (**b**). Comparison between the interpolation methods via objective metrics: SSIM (**upper**) and PSNR (**lower**).

The tests showed that the visual appeal produced by these two algorithms is similar for extra-urban routes, where the vehicle moves at a sustained speed and the number of discrepancies is relatively low. Conversely, the Laplace pyramid guarantees better results for urban environments. The difference between the two interpolations can be seen in Figure 5a. It is visible how the objects belonging to the first frame are completely visible within the interpolated frame.

Then, to objectively assess the interpolation quality, we have employed two standard perceptual metrics, namely the structural similarity index measure (SSIM) and the peak signal-to-noise-ratio (PSNR). These metrics are shown in Figure 5b: the SSIM and PSNR results are shown in the upper and lower graphs, respectively. From these outcomes, it is clearly visible that, on average, the Laplace pyramid outperforms the frame blending method, especially regarding the interpolation of the middle frames. The Laplace method also yields slightly higher PSNRs, thus confirming that the Laplace pyramid interpolation provides higher visual fidelity, especially in urban scenes where detail preservation is more critical.

#### 3.3.2. Frame Analysis

Considering the purpose of our application, it is essential to check the validity of the frames. Through an analysis conducted on the photospheres provided by Google APIs, two main types of errors can occur: frame missing and frame in a wrong way.

**Figure 6:**
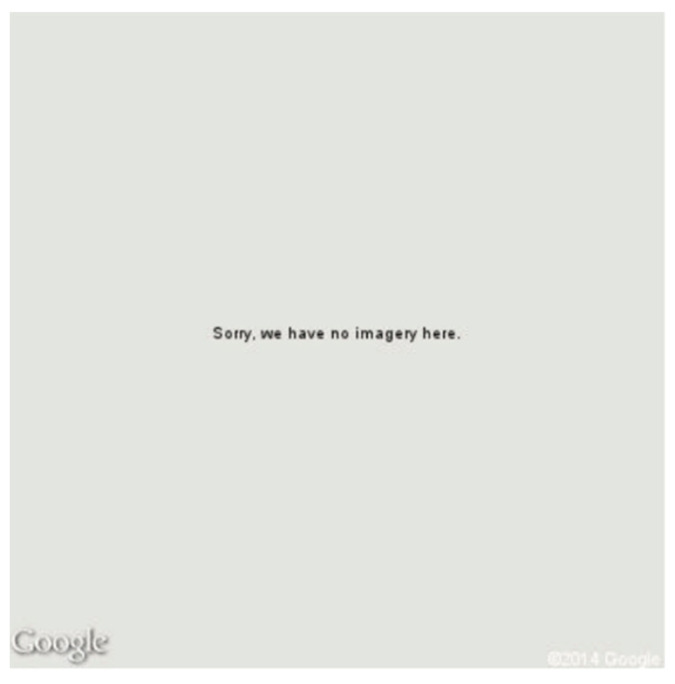
Examples of a missing frame.

**Figure 7:**
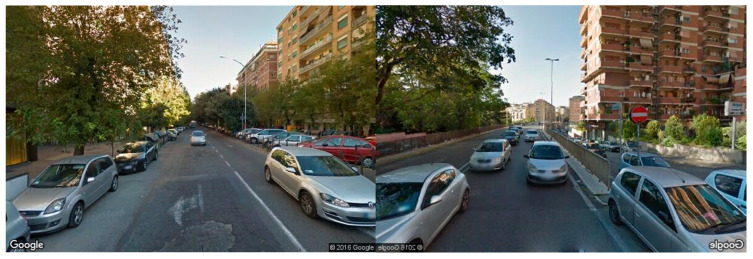
Examples of two frames (left and right) in the wrong way.

In a frame-missing scenario, Google does not have any picture for the requested coordinate. As shown in Figure 6, the API returns a grayscale image with an error message. To solve this issue, we compute the color histogram of each frame to detect if the frame turns out to be completely gray or not. If so, it can be considered as a missing image, and it is excluded from the video stream. In the frame in a wrong-way case, some roads are mapped only in one driving direction. Such a situation occurs when you ask Google for a set of images that must be oriented according to the direction of travel not traveled by a Google Car (a particular car that travels the unmapped roads taking photos in succession), resulting in a photo sequence apparently traveled in the opposite direction (Figure 7).

#### 3.3.3. Wrong-Way Detection Using CV Techniques

The detection of a frame in the wrong way requires a more sophisticated solution than the detection of a missing frame. This kind of frame causes a bad user experience, invalidating the produced output; therefore, it must be recognized and managed with an efficient pre-processing phase. To solve this issue, we developed a wrong-way detection algorithm based on two very well-known CV techniques: lane and car detection. In the following, we highlight the main implementation steps employed in our algorithm.

The rationale of the proposed lane detection method is based on two image processing techniques: Canny Edge Detection [49] and Hough Lines Transform [50]. Through the Canny Edge Detection, the algorithm can find edges in a grayscale image, returning a binary image where the positive values indicate the presence of edges. This technique is based on a thresholding approach with a local maximum to remove false positives.

**Figure 8:**
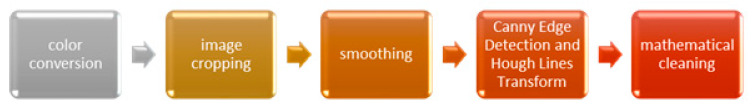
Block scheme of the entire proposed procedure for lane detection.

The proposed approach consists of taking the original picture and following these steps (see Figure 8):

*(i)*  *Color conversion*: we apply a color space transformation to convert the RGB picture into a grayscale image. The need to carry out this operation is attributable to the following reasons:
  - The lines and outlines do not undergo any alteration during this process;
  - The coloring of the elements does not add any relevant information content.

The conversion process involves a significant decrease in the data structure describing the resource, making a reduction from the three-dimensional to the two-dimensional plan.

**Figure 9:**
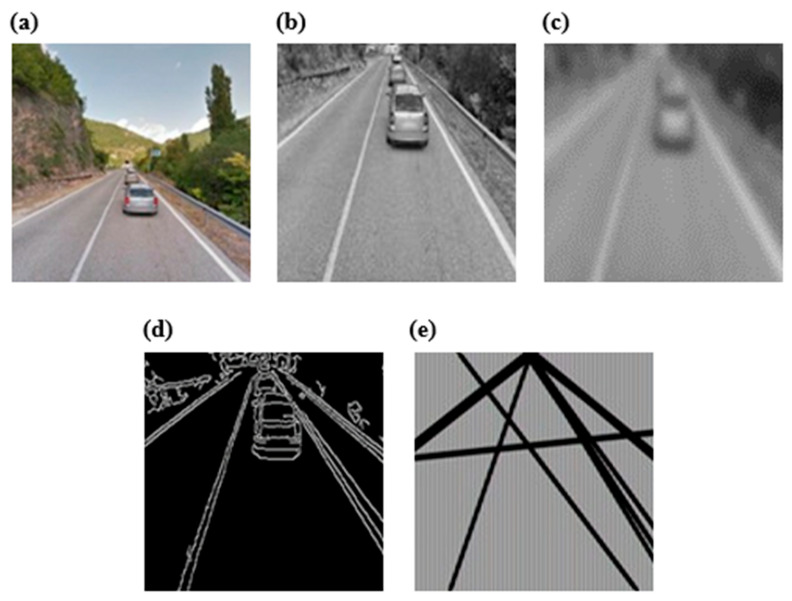
(**a**) Original image; (**b**) cropped image in grayscale; (**c**) smoothed image; (**d**) Canny Edge Detection; (**e**) Hough Lines Transform.

**Figure 10:**
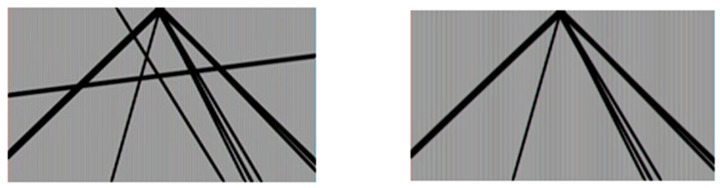
(**left**) Canny’s algorithm lines; (**right**) post-elaboration lines.

**Figure 11:**
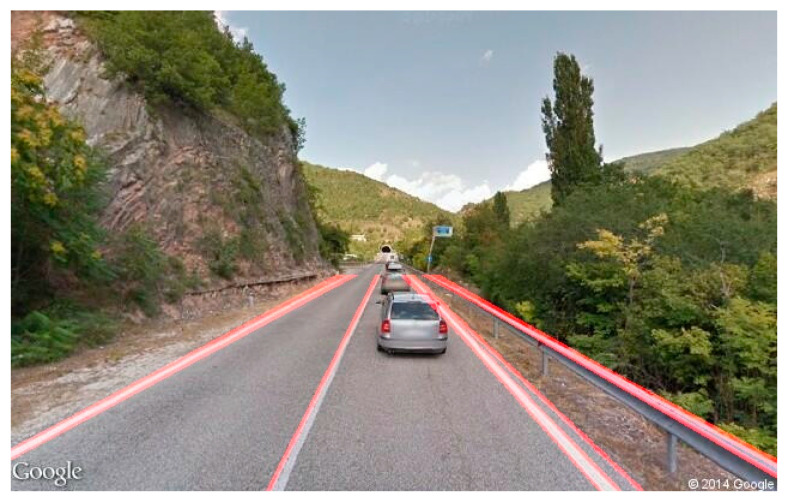
Example of lane detection applied on a real frame.

*(ii)*  *Image cropping:* if an analysis is carried out on the perspective related to the input image, it can be observed that the object sought within the task is always located at the lower end. This assumption is always true, as the position and inclination of the camera integrated with the Google Car is fixed and unchanged over time. It is therefore superfluous to examine all the information, which results in the following outcomes:
  - Introduce noise because the system may detect false positives (FPs), highlighting lanes in the absence of a road;
  - Involve a waste of computational resources since the road is always located at the lower end of the image;
  - Perform an analysis on the entire data structure doubling the information to be read and processed and the computational time.
*(iii)*  *Smoothing*: an operation of filtering on the image is necessary, called blur or smoothing, whose task is the reduction of the noise, since the images are characterized by both noise and visual details that are obviously useful only for improving the satisfaction perceived during viewing the video. A Gaussian-type filter was also used, represented by a kernel of dimensions 3 × 3 with σ = (5, 5).
*(iv)*  *Canny Edge Detection and Hough Lines Transform*: once the pre-processing phase has been completed, it is possible to carry out the technique that represents the heart of lane detection: the Canny edge detector algorithm (see Figure 9). Through its use, a domino transformation of the image is carried out, to change not only the content but also its meaning. While Canny’s input is represented by a raster image, i.e., expressed by a matrix whose cell value expresses the amount of brightness associated with a point, the output consists of an image of a Boolean matrix, having the same dimensions as the entrance structure. The meaning attributed to the single cell indicates whether it belongs to the contour, as detected using appropriate thresholds necessary for the algorithm. After applying the Canny edge detector algorithm, the Boolean matrix that is produced becomes the input of the Hough Line Transform, which examines the structure producing the lines recognized in it. As for Canny, a study of the threshold value was carried out, which was empirically fixed with a value of 150.
*(v)*  *Mathematical cleaning*: each line is analyzed mathematically, studying its slope. Once the previous phase has been completed, a set of straight lines extrapolated from the starting frame is available, but the elaborated product is not free from noise, since the straight lines obtained do not actually represent the road lines but instead represent any straight line passing through a contour detected by the Canny’s algorithm. This varies with the number of elements present in each frame, which varies according to the scenario in which the 360-photosphere was taken. Urban and congested areas will therefore produce higher noise than the rarely frequented extra-urban sections. A filter was then applied based on the following assumption: for a detected line to correspond to a road line, it must converge and intersect one or more of the sides of a rectangle placed in the center of the original image. The function of the rectangle is to represent the locus of the points of the plane where the vanishing point is free to oscillate, as this varies with the type of curve and with the inclination of the road depicted in the single frame. The choice of this geometric figure is due to its simplicity of implementation, as it can be described by the intersection of four straight lines. The dimensions of the rectangle are equal to 30% of the original size and fixed empirically after a study carried out on the available frames. Two suitable intervals have also been elaborated that filter the obtained lines, with one for each angle, empirically expressible by the values of the angular coefficient describing a straight line: m ∈ (−8, −0.75) ∨ (1, 6), see Figure 10 (left, right). Once the filtering is finished, it is necessary to prepare a method to bring the lines back to the frame, both for debugging reasons and for possible additional system functionality. It is sufficient to implement a function that considers both the relative position attributed to each single line and the transformation of the lines into segments (see Figure 11).

**Figure 12:**
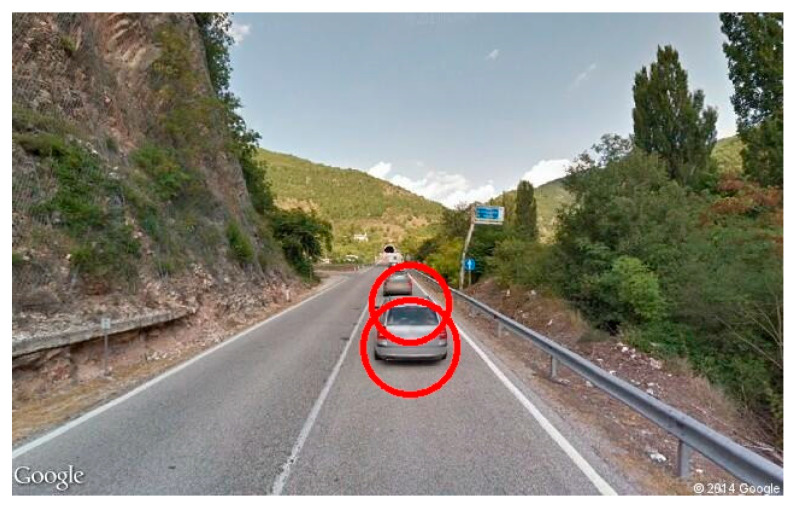
Example of car detection.

Then, the car detection has been realized through a standard object detection solution: via the Haar Feature-based Classifier Cascade (see Figure 12). The Haar Feature-based Cascade Classifier is a very effective object detection technique that exploits the machine learning technique known as prediction classification, from which it extracts a model composed of essential characteristics, called Haar-features. These are represented by small kernels used in the convolution phase and can be divided into three main categories, which are as follows:

- Edge features: features related to the edge.
- Line features: line features.
- Four-rectangle features: features based on four adjacent rectangles, to form a larger square.

For each feature, the kernel is broken down into rectangles, and its integral is calculated. The matching that will be calculated will no longer be based on arbitrary and numerous quantities of pixels but simply on a comparison of their areas, which are at most four, drastically reducing the computational cost associated with the operation. Once the features have been identified, they must be tested on each training image to understand which ones are relevant, keeping only those with a low error rate. The final classifier will be the sum of the different classifiers used, which are called weak as they would not be able to recognize objects if used individually. An important property of these classifiers is that they can be used sequentially if properly trained, hence the name Cascade Classifier. Creating a classifier is not a problem of simple resolution, but in the literature, two works emerged among others, namely the following:

- UIUC Image Dataset for Car Detection: a collection of images conducted by the University of Illinois at Urbana-Champaign [51].
- Database for vehicle detection: a work carried out by Alexander Gepperth in collaboration with the Honda Research Institute [52].

The first one is a study conducted between 2002 and 2004 which collected a quantity of photographs, relating to vehicles and not, and is useful for the training of a generic classifier for car detection. The dataset contains purely vehicles in use in American territories, between the late 90s and the early new millennium, and does not make any kind of distinction on the side shown by the cars. It also provides a consistent set of non-vehicle photographs, which is also useful in the training phase of the system, which can build a model starting from two sets: one showing positive examples and one for negative ones.

The second study was carried out in 2011 and represents the only research that has dealt with the classification of vehicles, such as recognizing the side shown. The dataset shows the negative elements, coming from the UICC research, and two elements for the positives, one of the UICC of about 500 images and one relating to the cut-out ROI (region of interest) consisting of about 16,000 images.

Finally, the produced algorithm consists of the following steps:

(i) Initially, it is necessary to recall the functions related to car and lane detection, which will return, respectively, a collection of vehicles and lines. In fact, it is possible to create a method that returns a Boolean value equal to true (false) if the scenario portrayed in the photograph is relative to a wrong (correct) path. The first phase of the algorithm is to examine the two basic cases:
  1. Empty car collection;
  2. Vehicles showing the rear only. This situation can represent several cases, which are illustrated considering a two-lane road:
    - The detected cars are located exclusively in the left lane;
    - The detected cars are located exclusively in the right lane. This case study includes two of them: one in each direction attributable to the left. The street could in fact be indiscriminately two-way or one-way;
    - There is at least one car per lane. The surveys induce the system to consider the carriageway characterized exclusively by a direction of travel, which is shared with the orientation of the camera.

It is not possible to say anything in the case in which all the cars have the front side, since it is possible to be in the second scenario (2. B), which would involve an indication of the relative answer. A method has been defined for carrying out the remaining checks as follows.

1. All the lanes show frontal cars: a positive result since it is the very definition of the wrong direction.
2. The front cars are in the lanes on the left and the rear ones on the right: this case coincides with what has been said about the front cars and represents one of the possible conformations of a road.
3. The front cars are in the left lanes, and cars have not been detected on the right.

It is necessary to define the method relating to the extraction of the lanes starting from the set of cars and lines. Lane detection may not produce the desired results: this can happen thanks to several factors, such as many elements placed above the lines on the ground or a shot characterized by low light or lines worn out by time and no longer perfectly visible. Being placed above a moving car, it is certain that there is at least one lane below it. This situation includes the following cases:

1. There is only one lane;
2. There are two lanes;
3. There are more than two lanes.

Although it is possible to have at least two lines, it is not certain that these delimit a lane. Indeed, the following is possible:

1. The lines obtained are equal to two and are too far from each other: in this case, it is assumed that there is at least one line between them; therefore, it is necessary to go back to the previous case;
2. The lines obtained are too close to each other: this situation may be caused by noise, and therefore it is unthinkable that a lane exists between them. This situation must be managed, and it is assumed, at least for the moment, that you have a method that does this.

Once the limit cases have been solved, it is necessary to describe the general behavior of the algorithm. Assuming you have both the method of filtering and sorting the lines, you can simply create a lane for each pair of lines identified by the available collection, subsequently inserting the car inside. It is important to remember that the lines are tilted due to the perspective associated with the camera lens. In fact, the lines that delimit a lane are not tangent but parallel to each other. It is therefore possible to calculate the real distance between them by considering the distance between the points of intersection with the lower end of the photograph, thus minimizing distortions due to perspective. The peculiarity of this algorithm lies in the fact of carrying out reconstructions in the absence of a correct output of the lane detection and correcting small anomalies of the car detection.

*(ii)*  The second phase of the algorithm therefore concerns the mathematical ordering of the detected lines: the insertion of the lines within the collection does not follow a precise order; therefore, they can be arranged in any order. To solve this issue, we use a square matrix, called the Position Matrix, whose cell [i] [j] indicates the position of the i-th line with respect to the j-th line. In this way, it is possible to extract an ordered list where each element is represented by the index associated with the line in question. The reordering of lines now becomes a trivial problem.
*(iii)*  In the third phase we dealt with the filtering of noise related to the lines: lane detection may produce more lines like each other, both in orientation and in position (called “noise”). This problem is solved by considering the distance between the segments of interest contained in the line itself. If this difference is less than the selected threshold, then the lines considered are similar and therefore belong to the same group.
*(iv)*  The 4th phase of the algorithm consists of inserting the cars in the lanes. The output provided by the car detector consists of a collection of car objects, which consist of the geometric description of a rectangle and a Boolean value indicating the side shown. It is set to true if the car shows the back side and is false otherwise.

By examining the collection of cars, it is possible to insert the single element within the correct lane, exploiting the methods already used to establish the relationship between two lines. This operation establishes the spatial relationship between a line and a point; therefore, it is sufficient for extracting a representative point of the car and inserting it inside the lane that includes it.

The two issues just raised can be solved separately as follows:

- We search for the point identifying the car (we consider the projection of the center of the figure on the lower side of the rectangle. In this way the possible errors are considerably reduced, as the spatial problem is reduced to one linear) or search for the lane containing the car.
- A lane contains a car if the car coding point is located to the left of the right line and to the right of the left line delimiting a lane.

*(v)*  The 5th and last part of the algorithm consists of a lane analysis. It is in fact possible that lane detection did not detect one or more lanes hosting one or more cars extracted from car detection: it is possible to solve this situation by introducing a check on insertion of the i-th vehicle, which if not inserted requires the allocation of a new lane.

#### 3.3.4. Implementation Details for Lane and Car Detection

To ensure reproducibility and clarify implementation choices, we provide here the specific parameters used for the computer vision components of our system. For lane detection, we employed a standard pipeline based on the Canny edge detector followed by the Hough Line Transform. The Canny thresholds were set to 50 (lower) and 150 (upper), using a Gaussian blur filter with a kernel size of 3 × 3 and σ = (5, 5) to reduce noise prior to edge detection. In the Hough Line Transform, we set the minimum line length to 40 pixels and the maximum allowed gap between line segments to 25 pixels, with a voting threshold of 150.

For car detection, we adopted the Haar Feature-based Cascade Classifier implemented in OpenCV. We used a pre-trained XML model derived from a combination of the UIUC Image Dataset for Car Detection and the Honda-Gepperth dataset, which includes labeled images of vehicles from multiple orientations (front, rear, and side). This choice allowed us to distinguish between front- and rear-facing vehicles, which is critical for wrong-way detection. Then, to mitigate false positives, particularly in dense urban environments, we introduced two key filtering steps: (*i*) lane–vehicle alignment check. Each detected vehicle is assigned to a lane using geometric intersection logic; inconsistent placements (e.g., floating vehicles or misaligned detections) are discarded. (*ii*) Symmetry-based consistency. When multiple vehicles are detected, the system verifies orientation symmetry across lanes (e.g., rear-facing vehicles in both lanes indicate correct direction), reducing misclassification. This parameter configuration provided a good trade-off between detection accuracy and computational efficiency and was selected after empirical tuning across both urban and rural test scenes. These settings are stable across typical Google Street View imagery, but the modular design allows for the future integration of deep-learning-based models (e.g., YOLO) when higher accuracy or GPU acceleration is available.

### 3.4. Performances of the Car and Lane Detection Algorithm

The goal of our work was not to create the best lane and car detection algorithm but to create a simulation system of road routes via video. We ran experiments on an Intel core i7, 3.1 GHz CPU equipped with 8 GB of RAM. The Google API has been used with the following limitations:

- Google Directions API: not high daily traffic (less than 2500 requests) must be guaranteed. More than two requests can be made per second.
- Google StreetView Image API: the photograph shown cannot exceed a specific resolution (600 × 400), and the number of requests must be below 25,000 per day.

The car detection has been realized through a standard Haar Feature-based Classifier using OpenCV. The performance of the proposed system is assessed using the following metrics: Precision (7) indicates the proportion of true positives among all predicted positives, while Recall (8), also known as Sensitivity, measures the proportion of actual positives that are correctly identified [53]. Accuracy (9) represents the overall correctness of the model:

(7) $$Precision=\frac{True\;Positive}{Predicted\;Positive}$$

(8) $$Recall=\frac{True\;Positive}{True\;Positive+False\;Negative}$$

(9) $$Accuracy=\frac{True\;Positive+True\;Negative}{Predicted\;Positive+Predicted\;Negative}$$

Note that True Positive (TP) derives from the fact that the model correctly classifies the positive samples, which is the case when positive samples are classified as positive. Conversely, True Negative (TN) corresponds to the fact that the model correctly classifies the negative samples, which is the case when negative samples are classified as negative [54].

We report the following results obtained in terms of accuracy, precision, recall, and execution times:

- Accuracy: 95.8%;
- Precision: 75%;
- Recall: 90%;
- Execution Time: 13.456 s per frame.

The algorithm can therefore keep track of lanes even when they are partially or completely invisible due to erosion or occlusion or when the lanes are not even painted on the road. However, the results of the experiment show that the proposed lane detection methods can work reliably but exhibit limited performance in terms of processing speed. As detailed before, the system operates as a proof-of-concept and was executed on standard consumer-grade hardware (Intel Core i7 CPU; 8 GB RAM) without GPU acceleration. Consequently, certain modules, particularly car and lane detection, require several seconds to process a single frame. This trade-off was intentional: the system was designed to prioritize correctness, modularity, and reproducibility over real-time performance. Let us now clarify the importance of this trade-off: the system’s current architecture was optimized for ease of deployment, maintainability, and cloud compatibility, rather than for high-throughput video processing. This makes it suitable for offline route analysis, route planning, or integration in web-based services where batch processing is acceptable and real-time constraints are relaxed.

## 4. Results

In this section, we will mainly focus on wrong-way detector tests. The technique used for the test is known as TDD (Test Driven Development). The identification of these tests is based on the idea of describing each time the functionality has not yet been foreseen by the system, verifying that the changes introduced have not altered the correct functioning of the previously described logic. Critical situations, which the system must be able to manage, have been conceived over time. Thanks to this type of approach, it is possible to accomplish the following:

- Fill any bugs introduced over time;
- Think in iterative steps;
- Establish various borderline cases, useful for ascertaining the validity of the algorithm itself.

To evaluate the performance and robustness of our system, we conducted experiments on a set of 24 route scenarios, carefully selected to represent a diverse range of road conditions and environments. The dataset includes the following:

- A total of 14 urban routes traversing densely built areas in cities such as Rome and Milan, characterized by high visual complexity, frequent intersections, and varied lighting conditions.
- A total of 6 suburban routes located in mixed residential and commercial areas with moderate traffic density and road signage.
- A total of 4 rural routes composed of long, low-traffic segments often lacking clear lane markings or proper lighting, which serve as stress cases for both detection and orientation estimation.

All real-world scenarios were generated using the Google Directions API and Google Street View Static API, which allowed us to collect street-level panoramas at controlled spatial intervals based on interpolated GPS polylines. In addition, we constructed a subset of synthetic scenarios by simulating idealized routes with uniform direction and spacing, designed to test the system’s interpolation smoothness and geometric consistency in isolation from visual clutter. Each route produced between 80 and 200 frames, depending on its length and curvature, resulting in a dataset of over 3500 annotated frames. This mixture of environments enabled us to validate the system across a broad spectrum of use cases, from structured city grids to visually sparse country roads, and under both ideal and challenging conditions such as occlusion, missing imagery, and ambiguous vehicle orientation. To simulate the scenarios, it was sufficient to instantiate a different number of car and line objects. The discussion of the tests is initially focused on roads formed by one or two lanes, postponing the remaining argument to a second phase. The following scenarios have been identified:

1. The whole scene occupies both lanes, and the vehicles show the rear side;
2. The whole scene occupies both lanes, and the vehicles show the front side;
3. Car detection was unable to detect vehicles;
4. There is only one front vehicle occupying the right lane;
5. There is only one front vehicle occupying the left lane;
6. Two cars were detected: one showing the front side and occupying the left lane, while the other is showing the rear side and occupying the right lane;
7. Prediction errors were made during car detection, which detected vehicles with different sides shown, located in the right lane. Most of the cars show the back side;
8. Prediction errors were made during car detection, which detected vehicles with different sides shown, located in the right lane. Most of the cars show the front side;
9. Prediction errors were made during car detection, which detected vehicles with different sides shown, located in the right lane. The number of vehicles showing the rear is approximately the same as the number showing the front.

The distribution of vehicles described in this way was combined with the following possible results of lane detection:

1. No significant lines were found;
2. A single line was identified within the frame;
3. The carriageway limiting lines have been identified, but not the lane separation;
4. The three lines delimiting the two existing lanes have been correctly identified;
5. Only the lines relating to the right lane have been identified;
6. The identification was successful, but it produced noise (lane detection may produce multiple similar lines; therefore, it confuses the lines of the lanes with other lines present in the images, both as orientation and as position) for all the detected lines.

**Figure 13:**
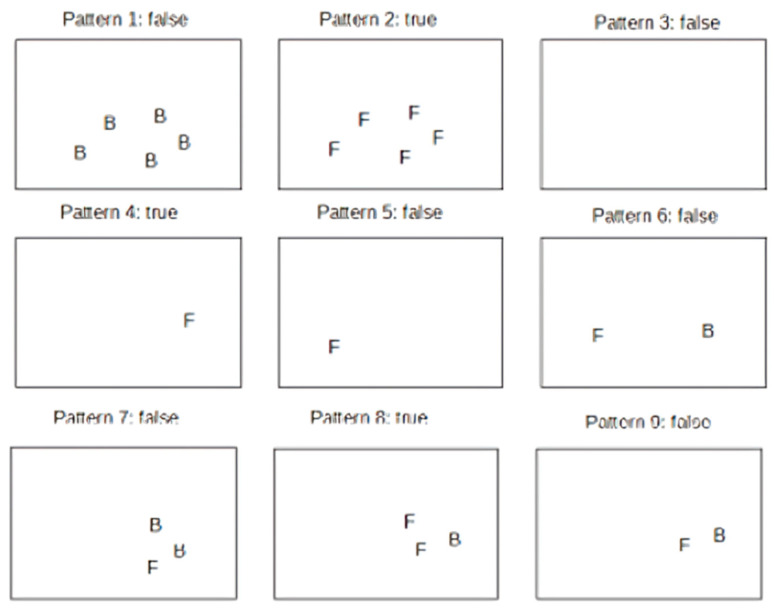
Scheme of the pattern followed by the tests for the simulation of vehicles. Each rectangle represents the instance of a frame, inside which vehicles are showing the back and front sides, respectively indicated by the letters B and F.

**Figure 14:**
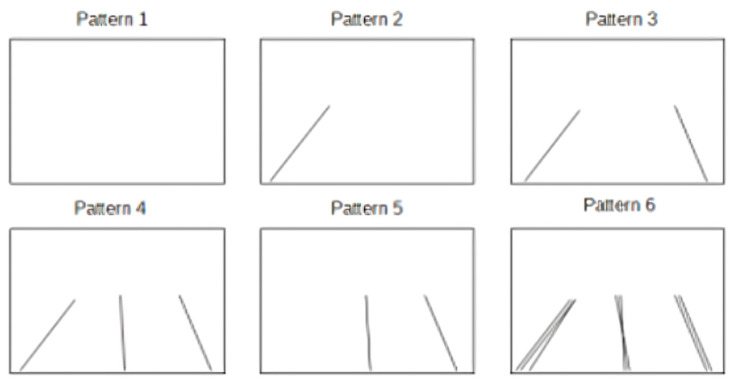
Scheme of the pattern followed by the tests for the simulation of the lines forming the lanes. Each rectangle represents the instance of a frame, inside which the lines detected by lane detection are reported.

Through this approach, we defined 54 tests during the entire implementation, such as to guide the correct development of the algorithm. It is possible to make a schematic representation of the tests performed; Figure 13 and Figure 14 show the scheme followed for the vehicles and the simulated lines, respectively.

**Figure 15:**
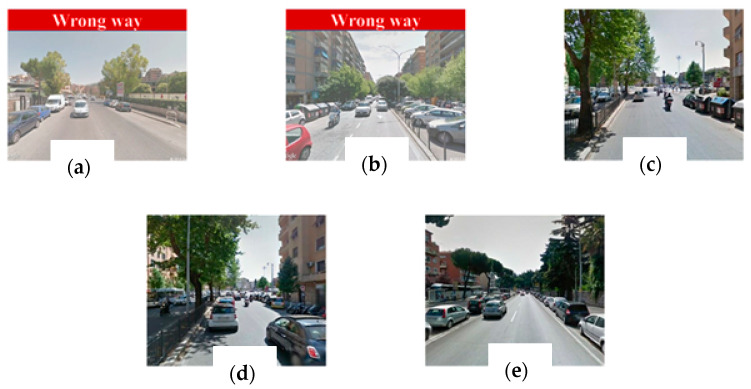
(**a**) Road in the wrong direction; (**b**) road in the wrong direction with partially visible lines; (**c**) regular road with invisible lines; (**d**) regular road with partially visible lines; (**e**) regular road with visible lines.

Analyzing the wrong-way frames in 85% of cases, it was observed that the lane and car detection algorithms worked correctly, and the wrong-way detector was able to define the wrong-way images by labeling them as such (see Figure 15). Here we can report the images of the most significant tests carried out.

**Figure 16:**
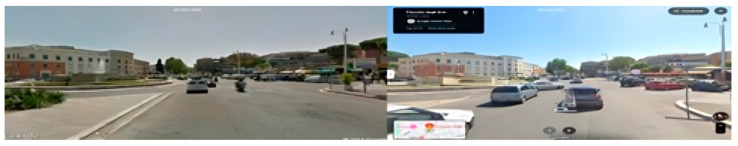
Side-by-side comparison of frame generation using our system (**left**) and Hyperlapse (**right**) at Piazzale degli Eroi, Rome.

To further validate the effectiveness of our proposed system, we finally conducted a comparison analysis with Hyperlapse, one of the most widely recognized tools for automated video generation from Google Street View imagery. While Hyperlapse offers excellent performance in terms of real-time rendering and responsiveness, it suffers from two key limitations: the inclusion of wrong-way frames due to lack of orientation filtering and the reliance on frame skipping, which often results in perceptually abrupt transitions. Conversely, our system focuses on orientation correction and perceptual smoothness through frame interpolation techniques. Frames from Hyperlapse were visually compared and found to have sharp perspective jumps and no intermediate smoothing, which degrades the viewing experience, especially at turns and intersections. We can see in Figure 16 that our system demonstrates frame alignment, correct orientation, and interpolation continuity under modest lighting conditions. Conversely, the Hyperlapse frame shows higher contrast and resolution but lacks orientation correction (as seen from the car turning diagonally), with no temporal smoothing between frames.

Our method, although slower (see below), provides a more natural and consistent visual flow. In terms of processing speed, our current implementation, executed on standard hardware without GPU acceleration, produced the following average execution times per module: (*i*) lane detection: ~7.2 s per frame; (*ii*) car detection (Haar Cascade): ~13.4 s per frame; (*iii*) wrong-way filtering + orientation correction: ~2.1 s per frame; and (*iv*) interpolation (Laplacian pyramid): ~0.4 s per frame. While this results in a total of ~23 s per frame, we stress that the system is intended as a cloud-based offline processing tool, not for real-time rendering. Performance optimizations, such as parallelization and GPU-based object detection (e.g., YOLOv8), are outlined in the discussion section as part of future work. Thus, while Hyperlapse excels in execution speed, our system offers greater semantic correctness, frame quality, and structural flexibility, especially for applications where video fidelity and correct orientation are critical.

Finally, the system can play a short video showing the path between two geographic locations. Here is a link to some videos obtained using the application in .AVI format that simulates a user’s guide: https://github.com/sp4telab/street-video (accessed on 15 July 2025). Currently, our current system is limited to detecting lanes and vehicles, as these were identified as the most critical elements for the specific task of detecting wrong-way frames and ensuring orientation correctness within Google Street View panoramas. This focused approach allowed us to develop a modular and computationally feasible prototype, while still addressing key challenges in route visualization. However, we acknowledge that this limited semantic scope represents a constraint on the system’s broader applicability. In real-world navigation and road understanding tasks, other visual elements—such as traffic signs, traffic lights, crosswalks, road surface types, and pedestrian zones—play an essential role in interpreting scene context and road semantics. In future versions of the system, we plan to expand the set of detectable features by integrating the following: (*i*) semantic segmentation models to extract multiple object classes from frames; (*ii*) lightweight detectors for traffic infrastructure, including signs and signals; (*iii*) road-type classifiers to distinguish between highways, local streets, and pedestrian areas. This expansion will not only improve scene understanding but also open opportunities for context-aware video rendering, such as emphasizing critical navigation points, highlighting hazards, or adapting video playback based on semantic road categories. By moving toward a richer and more diverse set of visual cues, the system can provide users with a deeper, more realistic understanding of the routes, bringing it closer to the capabilities of advanced driver-assistance systems (ADASs) and intelligent transportation platforms.

## 5. Conclusions

In this work, we proposed a modular, cloud-based system for generating smooth and immersive street-view route videos, integrating Google Maps APIs with image processing and computer vision techniques. The system addresses several limitations of existing tools—such as incorrect camera orientation, missing frames, and wrong-way images—through geometric correction and visual analysis powered by lane and car detection.

Our interpolation strategy, combining classical inbetweening techniques like frame blending and Laplace pyramid methods, offers a lightweight yet effective approach to simulating smooth video transitions without the need for high-performance computing resources. Quantitative evaluations using SSIM and PSNR metrics suggest that Laplace pyramid-based interpolation provides superior structural and perceptual quality, particularly in urban settings. The key innovation lies in the approach taken for frame alignment. The angle between each pair of frames was calculated using the cosine theorem and computer vision techniques, specifically lane and car detection. This meticulous frame alignment is crucial for constructing videos that maintain a consistent perspective, free from artifacts and errors. The methodology for identifying frames in the wrong direction showcases adaptability, providing reconstructions even in scenarios where lane detection outputs are absent or exhibit minor anomalies. The conducted tests, spanning both real-world and synthetic scenarios, have yielded promising results, affirming the robustness and efficacy of the proposed system. In addition, this method is computationally efficient and generally reliable for standard road geometries, particularly straight or gently curved routes. However, it may not account for several real-world challenges such as abrupt changes in road curvature (e.g., roundabouts; U-turns); GPS path irregularities due to mapping inconsistencies or GPS jitter; complex maneuvers (e.g., sharp turns; switchbacks); and temporal smoothness, which is important for generating perceptually coherent video sequences.

## 6. Limitations and Future Work

Future versions of our system could surely benefit from the integration of techniques such as the following: (*i*) visual odometry: leveraging image content across frames to refine orientation and translation vectors, enabling a more realistic reconstruction of camera motion; (*ii*) RNN-based smoothing: applying recurrent neural networks (e.g., LSTM or GRU models) to learn consistent orientation transitions across a sequence of frames, reducing noise from both interpolation artifacts and inconsistent GPS data; (*iii*) sensor fusion approaches, which may combine GPS, IMU data (if available), and image features for higher-fidelity localization. These enhancements would not only improve the visual consistency of generated videos but also make the system more robust to irregular paths, GPS noise, and diverse road types, extending its applicability to more complex navigation environments.

However, we acknowledge that certain system components, such as object detection and frame analysis, currently rely on static classifiers and manually tuned rules. As part of the research’s forward-looking perspective, future developments are outlined, prioritizing the enhancement of video fluidity through advanced inbetweening techniques and the incorporation of modern ML/DL solutions to further refine the wrong-way detector. These initiatives underscore the commitment to continuous improvement and staying at the forefront of technological advancements. While our internal evaluations—such as the wrong-way detection tests and perceptual quality analysis of interpolated frames—have demonstrated promising results, we acknowledge a key limitation in terms of external validation. Given the specialized nature of our pipeline, which relies on Google Street View photospheres instead of real-time video feeds or standard driving datasets, benchmarking against widely used datasets (e.g., KITTI, Cityscapes, and BDD100K) is not directly applicable.

Nonetheless, we agree that incorporating baseline comparisons with publicly available datasets—even if only partially aligned with our use case—would greatly strengthen the scientific rigor of our evaluation. As part of future work, we plan to identify adaptable benchmark datasets that include similar urban scene elements (e.g., road structure, vehicles, and orientation shifts) and to develop a custom evaluation set derived from Street View data annotated with ground truth for orientation and object detection tasks. Finally, we aim at exploring transferability of pretrained models on benchmark datasets to our domain, evaluating performance drops and potential fine-tuning strategies. This direction will help validate the system’s effectiveness in a broader context and facilitate more meaningful comparisons with state-of-the-art solutions in computer vision for road understanding and video-based navigation.

A promising direction for future work is the integration of continual learning in the object detection modules. This would enable the system to adapt over time to new visual contexts, road conditions, vehicle types, and environmental changes (e.g., seasonal or weather variations). Continual learning would also mitigate issues related to domain drift and allow the classifier to improve incrementally as more data is collected during system use. By employing modern DL frameworks (e.g., YOLOv8+, EfficientDet, or Vision Transformers) in conjunction with continual learning paradigms (such as Elastic Weight Consolidation or replay-based methods), the detection modules could evolve to deliver increasingly accurate and robust performance without requiring full retraining. In future iterations, we plan to explore the following: (*i*) online fine-tuning of detection networks based on user feedback or unsupervised cues; (*ii*) integration of edge/cloud-based adaptive learning strategies to balance performance and resource usage; (*iii*) expansion of the CV pipeline to include semantic segmentation of traffic signs, pedestrian crossings, and road types.

In summary, this work lays a solid foundation for automated route video generation that seamlessly integrates Google Maps and street view videos, providing users with a visually engaging and accurate representation of road paths. The incorporation of mathematical principles and cutting-edge computer vision technologies stands out as a critical path forward to achieving a scalable, adaptive, and intelligent visual navigation system.

## Data Availability

The original contributions presented in this study are included in the article. Further inquiries can be directed to the corresponding author.
